# A helical perylene diimide-based acceptor for non-fullerene organic solar cells: synthesis, morphology and exciton dynamics

**DOI:** 10.1098/rsos.172041

**Published:** 2018-05-02

**Authors:** Li Chen, Mingliang Wu, Guangwei Shao, Jiahua Hu, Guiying He, Tongle Bu, Jian-Peng Yi, Jianlong Xia

**Affiliations:** 1 School of Chemistry, Chemical Engineering and Life Science, Wuhan University of Technology, No. 122 Luoshi Road, Wuhan 430070, People's Republic of China; 2 State Key Laboratory of Advanced Technology for Materials Synthesis and Processing, Wuhan University of Technology, No. 122 Luoshi Road, Wuhan 430070, People's Republic of China

**Keywords:** perylene diimide-based acceptor, organic solar cells, synthesis, exciton dynamics

## Abstract

Helical perylene diimide-based (hPDI) acceptors have been established as one of the most promising candidates for non-fullerene organic solar cells (OSCs). In this work, we report a novel hPDI-based molecule, hPDI_2_-CN_2_, as an electron acceptor for OSCs. Combining the hPDI_2_-CN_2_ with a low-bandgap polymeric donor (PTB7-Th), the blending film morphology exhibited high sensitivity to various treatments (such as thermal annealing and addition of solvent additives), as evidenced by atomic force microscope studies. The power conversion efficiency (PCE) was improved from 1.42% (as-cast device) to 2.76% after thermal annealing, and a PCE of 3.25% was achieved by further addition of 1,8-diiodooctane (DIO). Femtosecond transient absorption (TA) spectroscopy studies revealed that the improved thin-film morphology was highly beneficial for the charge carrier transport and collection. And a combination of fast exciton diffusion rate and the lowest recombination rate contributed to the best performance of the DIO-treated device. This result further suggests that the molecular conformation needs to be taken into account in the design of perylene diimide-based acceptors for OSCs.

## 1. Introduction

Solution-processed bulk heterojunction organic solar cells (OSCs) have drawn extensive attention due to their fascinating advantages, such as low-production cost, light weight and potential flexibility [1–5]. Considerable efforts have been dedicated to material design, device optimization and mechanism studies, and this field has achieved great progress during the past decades [6–10]. Fullerene and its derivatives with high electron affinity and high mobility have made significant contributions to this field in this process [11–15]. And nowadays, OSCs based on fullerene acceptors have achieved superior device performance with power conversion efficiency (PCE) over 10% [16–18]. However, several intrinsic defects, such as relatively weak absorption in the visible region and difficulty in molecular structural modification, and fixed electronic structure, present serious barriers for their further improvement [19–22]. Recently, non-fullerene small molecular acceptors have attracted increasing interest because they exhibit many important advantages towards their fullerene counterparts, such as stronger absorption in the visible region, more flexible structural modification, and tunable optical and electronic properties [23–26].

**Scheme 1.**
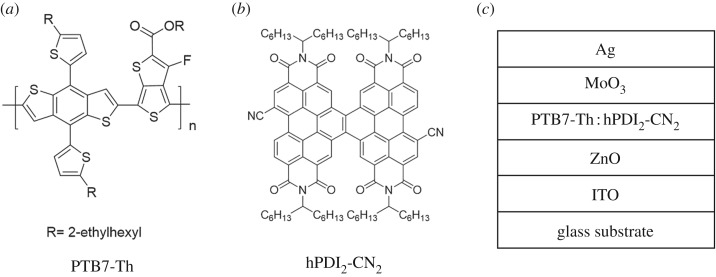
Chemical structures of PTB7-Th (*a*) and hPDI_2_-CN_2_ (*b*). (*c*) Inverted device structure.

Perylene diimide (PDI)-based small molecule is one of the most extensively investigated non-fullerene acceptors in recent years. This kind of n-type organic semiconductors holds many merits for photovoltaic devices, such as ease of synthesis and reproduction, high thermal and chemical stability, excellent electron-accepting ability and high electron mobility [27–30]. These highly desirable merits make PDI and its derivatives very suitable to serve as electron acceptor for OSCs. Although notable improvements have been achieved in recent years for PDI-based OSCs, there are still some issues to be addressed for this kind of acceptor [31–33]. Traditional PDI-based molecules suffer from serious self-aggregation that can be ascribed to their rigid planarity. In this case, the strong aggregation will lead to large crystalline domain size, which can result in large phase separation. It is well known that large phase separation is detrimental for device performance, since it is not favourable for exciton diffusion (ED) and dissociation [34,35]. Therefore, it is important to depress the aggregation while maintaining charge transport properties of PDI-based acceptors. Recently, a number of novel molecule design strategies have been reported and various novel PDI-based molecules with different conformations have been explored as electron acceptors in OSCs. Helical PDI (hPDI) is one of the most outstanding electron acceptor materials among the numerous PDI-based materials [36–38]. With a twisted chemical structure, the issue of self-aggregation for hPDI and its derivatives has been well addressed without weakening their carrier transport performance. The introduction of substituents at bay regions is a favourable modification strategy to enhance the performance of PDI-based devices. In this strategy, some electron-withdrawing groups, such as F, CN can bring about increased electron affinity which results in lower lowest unoccupied molecular orbital (LUMO) level and superior electronic properties.

In this contribution, a novel hPDI molecule, hPDI_2_-CN_2_, has been reported and explored as electron acceptor in OSCs. As shown in scheme 1, hPDI_2_-CN_2_ consists of two PDI units and two cyano groups at the bay regions. The synthesis details and characterization results are summarized in electronic supplementary material, figures S1 and S4. The microscopic morphology of PTB7-Th : hPDI_2_-CN_2_ blending film exhibits high sensitivity to various treatments, such as thermal annealing and additive treatments. Encouraged by the characteristics, we conducted further work on tuning the morphology in order to improve the device performance. Enabled by simple thermal annealing, the PCE of the device based on PTB7-Th : hPDI_2_-CN_2_ has been remarkably enhanced from 1.42% to 2.76%. Impressively, with 0.5% 1,8-diiodooctane (DIO) treatment, the PCE has been further promoted to 3.25% with an open circuit voltage (*V*_oc_) of 0.545 V, a short-circuit current density (*J*_sc_) of 9.77 mA cm^−2^, a fill factor (FF) of 61.1%. This work demonstrates that helical conformation is a promising archetype for developing highly efficient PDI-based acceptors and that careful morphology optimization is crucial for improving performance of hPDI-based non-fullerene OSCs.

## 2. Experimental details

### 2.1. Materials

All chemicals used during the device fabrication, unless otherwise specified, were purchased from Sigma-Aldrich. PTB7-Th was obtained from Luminescence Technology Corporation. These materials were used as received without further purification. The synthesis of hPDI_2_-CN_2_ and related characterizations were summarized in the electronic supplementary material.

### 2.2. Device fabrication

The pre-patterned (sheet resistance, 15 Ω/sq) ITO-glass substrates were sequentially cleaned in ultrasonic bath with detergent (Alconox Inc.), deionized water, acetone and isopropanol. The oven-dried substrates were then treated by an oxygen plasma (180 W) for 5 min. The inverted devices were fabricated with the structure of ITO/ZnO/active layer/MoO_3_/Ag. The ZnO precursor solution (110 mg ml^−1^) was prepared by dissolving 0.22 g ZnAc_2_·2H_2_O in 2 ml 2-methoxyethanol and 0.056 ml ethanol amine and then stirred for at least 24 h. The solution was filtered with polyether sulfone filters. The ZnO precursor solution was spin-coated onto ITO substrate with spinning rate of 5000 r.p.m. for 60 s and the thickness was approximately 32 nm. The as-cast film was then annealed at 150°C for 60 min to form a compact ZnO layer. PTB7-Th and hPDI_2_-CN_2_ were mixed in chlorobenzene with various mass ratios. The overall concentration was 25 mg ml^−1^ and the solution was heated at 60°C and stirred overnight. The active layer was fabricated by spin-coating the prepared solution onto ZnO layer with a spinning rate of 1500 r.p.m. and spinning time of 50 s, which was followed by thermal annealing treatment at 160°C for 40 min. A MoO_3_ (8 nm) layer and an Ag layer (100 nm) electrode were sequentially deposited by thermal evaporation using a shadow mask under a vacuum of less than 1.0 × 10^−4^ Pa. The active area of the device, defined by the overlap region of ITO and Ag electrodes, was 0.0625 cm^2^.

### 2.3. Characterizations

UV–Vis and photoluminescence (PL) spectra were recorded by Shimadzu UV3600 spectrophotometer and Zolix Flex One FX1-MPL500, respectively. Cyclic voltammetry (CV) pattern was measured by CHI660E Electrochemical Workstation. Film thicknesses were determined by Bruker DektakXT Stylus Profiling System. Microscopic surface morphology measurements were conducted on Multimode 8 atomic force microscope (AFM, Bruker, USA). Density functional theory (DFT) calculations were performed by using the Gaussian 09 software package at the B3LYP/6-31G(d) level. The alky chains were replaced by methyl groups to reduce the computation cost. All the optimized ground-state structures were shown to be minima by the absence of imaginary frequencies.

Current density–voltage (*J*–*V*) characteristics were measured using a Keithley 2400 source measure unit. The photocurrent was measured under AM 1.5 G illumination of 100 mW cm^−2^ provided by a Newport Solar Simulator (Model 94023A-U, class AAA solar simulator). Light intensity of the simulator was calibrated with an NREL-certified standard silicon reference cell and a readout meter. External quantum efficiency (EQE) was determined by an EQE system (Zolix, China).

Transient absorption (TA) spectroscopy was performed on a commercial femtosecond pump-probe system (TA Spectrometer, Newport Corporation). Laser pulses at 1040 nm with less than 400 fs duration were generated by a 200 kHz amplified laser system (Spirit 1040-8-SHG, Newport Corporation). The probe beam was a white light continuum beam spanning 500–950 nm spectral region, created by focusing a fraction of the 1040 nm fundamental output onto a YAG crystal. The rest of the output generated the pump pulses at 520 nm by second harmonic generation. The pump-probe delay was controlled by a mechanical delay stage. Excitation fluence in each measurement was 30 µJ cm^−2^.

## 3. Results and discussion

**Figure 1.**
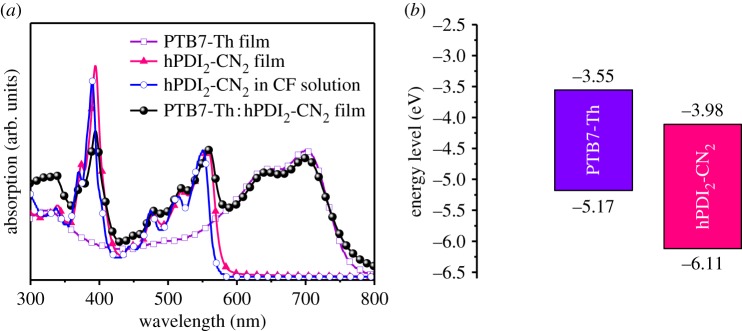
(*a*) The absorption spectra of PTB7-Th film (thickness, 120 nm, purple open squares), hPDI_2_-CN_2_ in dilute CF solution (concentration, 5 × 10^−6^ mol l^−1^, open circles) and film states (thickness, 80 nm, solid triangles), and PTB7-Th : hPDI_2_-CN_2_ blending film (thickness, 100 nm, 1 : 2.5, w/w, solid spheres). (*b*) The energy level diagram of PTB7-Th and hPDI_2_-CN_2_.

Figure 1*a* shows the UV–Vis absorption spectra of PTB7-Th film, hPDI_2_-CN_2_ in dilute chloroform (CF) solution and thin-film states, and the blend thin film. The absorption spectrum of PTB7-Th (scheme 1) film exhibits two major absorption bands at around 641 and 698 nm, which is consistent with those previously reported [18,31]. Broad absorption in the UV–Vis region is observed for hPDI_2_-CN_2_ in dilute CF solution with a sharp onset at approximately 575 nm and several major peaks lying around 337 nm, 443 nm, 474 nm, 514 nm and 550 nm, respectively. Compared with hPDI_2_-CN_2_ in CF solution, the absorption spectrum of spin-coated hPDI_2_-CN_2_ film (80 nm) shows approximately 8 nm red-shift while maintaining the spectral shape. This phenomenon suggests relatively low degree of self-aggregation and intermolecular interactions for hPDI_2_-CN_2_ film, which probably benefits from its helical conformation (as shown in electronic supplementary material, figure S4) that remarkably reduces π-electron conjugation. The absorption onset of hPDI_2_-CN_2_ film is observed at 583 nm, corresponding to an optical bandgap of 2.12 eV. The hPDI_2_-CN_2_ film shows a relatively high absorption coefficient (*ε*) of 6.8 × 10^4^ cm^−1^. Complementary absorption spanning from 300 to 800 nm would be achieved by incorporating PTB7-Th with hPDI_2_-CN_2_, which is desirable for the light harvesting and exciton generation of the photovoltaic devices.

The electrochemical property of hPDI_2_-CN_2_ was measured by CV method. As shown in electronic supplementary material, figure S6, the onset of the reduction potential (*φ*_red_) of hPDI_2_-CN_2_ is −0.25 V. Therefore, the highest occupied molecular orbital (HOMO) and LUMO energy levels, were calculated to be −6.11 eV and −3.98 eV, respectively (figure 1*b*). The experimental HOMO and LUMO levels are in accordance with those obtained from theoretical calculation (HOMO −6.41 eV, LUMO −4.03 eV and bandgap 2.38 eV) as summarized in electronic supplementary material, table S1. The HOMO and LUMO energy levels of hPDI_2_-CN_2_ are comparable to those of fullerene derivatives, such as [6,6]-phenylC61 (or C71)-butyric acid methyl ester (PC_60_BM or PC_71_BM), suggesting its potential as an electron acceptor [14,17]. As shown by the thermogravimetric analysis result in electronic supplementary material, figure S7, a relatively high decomposition temperature (*T*_d_) of 384°C is observed for hPDI_2_-CN_2_, manifesting high thermal stability.

**Figure 2.**
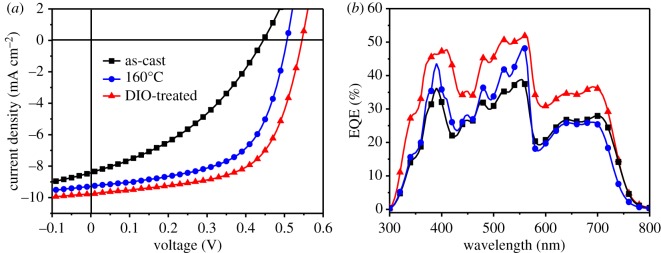
The *J*–*V* curves (*a*) and the EQE results (*b*) for PTB7-Th : hPDI_2_-CN_2_ solar cells based on different treatment conditions.

As shown in scheme 1*c*, the photovoltaic performance was characterized by an inverted configuration of ITO/ZnO (32 nm)/PTB7-Th : hPDI_2_-CN_2_ (100 nm)/MoO_3_ (8 nm)/Ag (100 nm), where ZnO and MoO_3_ served as electron transporting layer and hole transporting layer, respectively. Considerable efforts have been devoted to device parameter optimization, such as temperature and time modulation of thermal annealing, as well as additive treatments. When the blend film was annealed at 100°C, the devices showed remarkable increased FF of 51.8% compared to the value at 80°C (FF = 38.3%) as shown in electronic supplementary material, table S2. As the annealing temperature was increased to 160°C, the devices showed further-enhanced PCE of 2.45%. It is noted that the optimal D–A ratio is 1 : 2.5 and the optimized thermal treatment condition is annealing at 160°C for 40 min. The device parameters are summarized in table 1 and the corresponding current density–applied voltage (*J*–*V*) characteristics of the solar cells are shown in figure 2*a*. The as-cast device exhibits a relatively low PCE of 1.42% with an open circuit voltage (*V*_oc_) of 0.445 V, a short-circuit density (*J*_sc_) of 8.42 mA cm^−2^ and a FF of 38.0%. Compared with the as-cast device, a remarkable PCE enhancement of 94.4% is achieved for the thermal-annealed device.

**Table 1.** Summary of device parameters of PTB7-Th : hPDI_2_-CN_2_ solar cells based on various treatments.

device	*V*_oc_ (V)	*J*_sc_ (mA cm^−2^)	FF (%)	PCE (%)^a^	PCE_max_(%)
as-cast	0.415 ± 0.03	8.22 ± 0.2	35.0 ± 3	1.19 ± 0.13	1.42
160°C–40 min	0.486 ± 0.02	9.09 ± 0.2	56.7 ± 2	2.50 ± 0.26	2.76
DIO-treated^b^	0.535 ± 0.01	9.57 ± 0.2	60.1 ± 1	3.08 ± 0.17	3.25

^a^Statistical results obtained from 10 individual devices.

^b^The optimal amount of DIO was 0.5% (vol%).

Based on this optimized condition that the annealing temperature of 160°C for 40 min, we then modulated D–A mass ratios, such as 1 : 1, 1 : 1.5, 1 : 2.0, 1 : 2.5, 1 : 3.0 and 1 : 4.0. As shown in electronic supplementary material, figure S11 and table S3, an enhanced PCE of 2.76% was obtained with a D–A ratio of 1 : 2.5. The device parameters were shown as follows: *V*_oc_ = 0.506 V, *J*_sc_ = 9.29 mA cm^−2^ and FF = 58.7%.

Next, the influence of additive treatments on the device performance was further investigated. In fact, we have evaluated four high-boiling-point solvent additives: DIO (332°C), 1-chloronaphthalene (CN, 260°C), 1-methyl-2-pyrrolidinone (NMP, 203°C) and dimethyl formamide (DMF, 153°C). The *J*–*V* results for the devices based on different solvent additives were shown in electronic supplementary material, figure S12 and the corresponding technical parameters were collected in electronic supplementary material, table S4 for clear comparison. Among the four additive-treated devices, the best performance was obtained by the DIO-based device. Besides, the device optimization with a combination of DIO treatment and thermal annealing is described in detail in electronic supplementary material, figures S14 and S15 and table S5. As depicted in figure 2*a*, the device treated with 0.5% DIO (vol%) exhibits a further-enhanced PCE of 3.25% with the following device parameters (shown in table 1): *V*_oc_ = 0.545 V, *J*_sc_ = 9.77 mA cm^−2^ and FF = 61.1%. It is worth mentioning that this FF value is at relative high level for PDI-based non-fullerene solar cells [28,36,39–41].

The EQE results of the as-cast, thermal-annealed and DIO-treated devices are shown in figure 2*b*. All of the three devices show broad EQE spectra spanning from 300 to 800 nm, which is similar to the absorption spectra. The lowest EQE value of 39% (at approx. 553 nm) is obtained for the as-cast device, while a higher performance of 48% (at approx. 559 nm) is achieved for the device based on annealing treatment. Obviously, the highest value of 52% (at approx. 561 nm) is observed for the device with DIO treatment.

In order to investigate the exciton-dissociation efficiency, we have carried out PL spectra measurements for pure PTB7-Th film, pure hPDI_2_-CN_2_ film and PTB7-Th : hPDI_2_-CN_2_ blend films with various treatments. As shown in electronic supplementary material, figure S17, the pure PTB7-Th film (approx. 80 nm) and hPDI_2_-CN_2_ film (approx. 80 nm) exhibit strong emissions, while extremely weak emission behaviours were observed for the blend films. By comparing the intensity contrast, the fluorescence quenching efficiencies are estimated to be 92%, 95% and 97% for as-cast, thermal-annealed and DIO-treated devices, respectively. The highest quenching efficiency indicates the best exciton dissociation, almost complete quenching, at donor–acceptor interfaces for the DIO-treated blend, which can be responsible for the highest *J*_sc_, FF and EQE achieved by the DIO-treated devices.

The carrier transporting property was characterized by space-charge limited current method. The hole- and electron-only devices were fabricated with the structures of ITO/PEDOT : PSS/active layer/MoO_3_/Ag and ITO/ZnO/active layer/LiF/Al, respectively. As shown in electronic supplementary material, figure S18 and table S6, the hole and electron mobility values of as-cast PTB7-Th : hPDI_2_-CN_2_ blend film were estimated to be 6.0 × 10^−5^ cm^2^ V^−1^ s^−1^ and 9.0 × 10^−5^ cm^2^ V^−1^ s^−1^, respectively. For the thermal-annealed blend film, a higher hole mobility of 1.9 × 10^−4^ cm^2^ V^−1^ s^−1^ was obtained. Impressively, the electron mobility of 1.6 × 10^−3^ cm^2^ V^−1^ s^−1^ was achieved for thermal-annealed blend film, which was superior to that of as-cast film, thus facilitating exciton transport. For the DIO-treated blend film, a hole mobility of 4.3 × 10^−4^ cm^2^ V^−1^ s^−1^ and an electron mobility of 4.0 × 10^−4^ cm^2^ V^−1^ s^−1^ were obtained. With respect to the ratio of hole/electron mobility (*μ*_h_/*μ*_e_), the *μ*_h_/*μ*_e_ for the as-cast and annealing-treated films are both less than 1.0, suggesting hole mobility is much lower than that of electron mobility. By contrast, the *μ*_h/_*μ*_e_ for the DIO-treated blend film is 1.08, indicating superior balanced carrier transport property. This balanced transport behaviour will play a crucial role in the charge collection.

**Figure 3.**
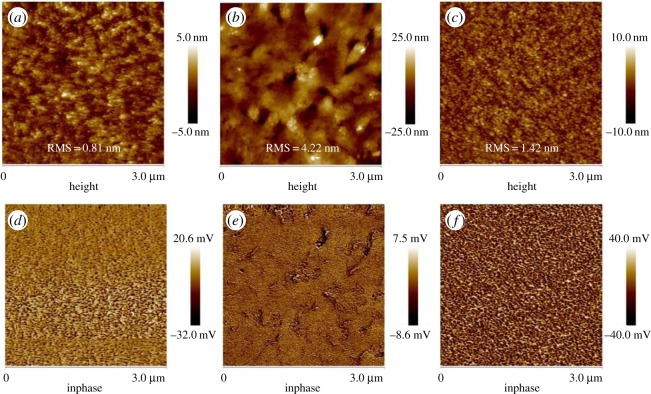
Atomic force microscope height images (*a–c*) and corresponding phase images (*d–f*) for the blend films with different treatments: as-cast, thermal annealing treatment and DIO treatment. The scanning size is 3 × 3 µm^2^.

The microscopic morphologies of active layers with different treatments were characterized by AFM. Atomic force microscope height images and corresponding phase images of as-cast, thermal-annealed and DIO-treated films are shown in figure 3. For the as-cast film, as shown in figure 3*a*, smooth and uniform topography with the lowest root mean square (RMS) roughness of 0.81 nm is observed, which indicates excellent miscibility between PTB7-Th and hPDI_2_-CN_2_. Furthermore, it is worthwhile to mention that no evident large aggregations are observed for the as-cast blend film, demonstrating helical conformation is highly beneficial for depressing aggregation. Heterogeneous phase separations with various domain sizes are observed from the phase image (figure 3*d*) of the as-cast film. This non-uniform phase separation is probably the origin of the lowest PCE for the as-cast devices. As displayed in figure 3*b*, the annealed film tends to form large crystalline domains with a dramatical increment of RMS roughness to 4.22 nm, demonstrating a rough surface morphology. This phenomenon might be ascribed to the crystallization of the blend film due to the low glass transition temperature (*T*_g_) of PTB7-Th (129.5°C) [42]. And this crystallization would be the origin of significant electron mobility improvement for the annealed blend film as discussed above. In addition, as displayed in figure 3*e*, no evident phase separation is observed from the phase image. With regard to the DIO-treated blend film (depicted in figure 3*c*), a relatively low RMS value of 1.42 nm is achieved, indicating the positive role of DIO treatment in suppressing self-aggregation effects of hPDI_2_-CN_2_. Moreover, according to the phase image shown in figure 3*f*, excellent homogeneous phase separation with two inter-penetrating mesh-like networks is observed. Moreover, the phase domain size is within tens of nanometres, which is highly desirable for ED and charge separation. Small domain sizes are beneficial for efficient exciton dissociation because the diffusion length of excitons is typically 5–20 nm. Therefore, with a combination of good morphology and suitable phase separation, the best performance can be achieved by the DIO-treated device.

**Figure 4.**
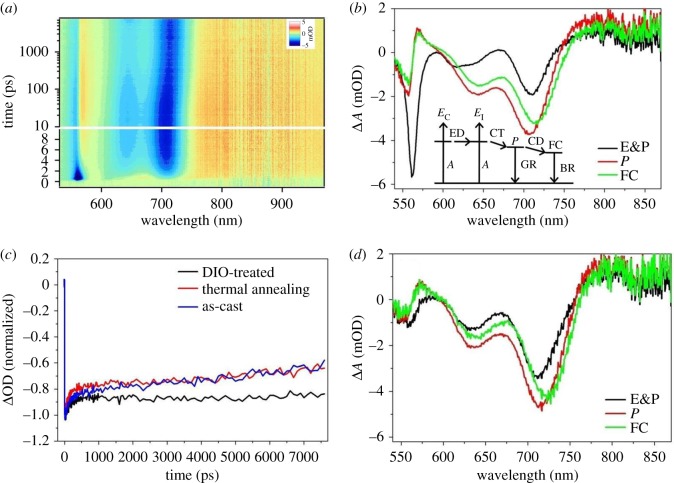
(*a*) The TA result of DIO-treated device shown in a pseudo-colour plot. (*b*) The TA global fitting results of the DIO-treated hPDI_2_-CN_2_/PTB7-Th device. The photovoltaic conversion process is depicted in the inset. The related states and processes are denoted as follows: *E*_C_, exciton in the crystalline domain; *E*_I_, exciton at the interface; *P*, polaron; FC, free carrier; *A*, absorption of photon; ED, exciton diffusion; CT, charge transfer; CD, charge dissociation; GR, geminate recombination; BR, bimolecular recombination. (*c*) The representative kinetics at 710 nm for the three devices. (*d*) The TA global fitting results of the as-cast PTB7-Th : hPDI_2_-CN_2_ device.

Transient absorption spectroscopy measurements were carried out to deeply understand the dependence of exciton dynamics on film microscopic morphology. By using the TA spectroscopy, we further demonstrated the differences of charge generation and recombination processes for the three devices: as-cast, thermal-annealed and DIO-treated devices. The detailed data, including their pseudo-colour plot, global fitting results and representative kinetics, are shown in electronic supplementary material, figure S19. The pseudo-colour plot of DIO-treated device is shown in figure 4*a*. At the beginning, the approximately 560 nm region is dominated by bleaching signal, corresponding to the population of hPDI_2_-CN_2_ exciton. The polaron signal of hPDI_2_-CN_2_ then builds up at approximately 570 nm. The approximately 640 nm and 710 nm regions are dominated by the bleaching signal of PTB7-Th while the region above 750 nm is dominated by the signal of PTB7-Th polarons. The polaron signal generates within the instrument response time and its intensity continues to increase until approximately 10 ps. This is attributed to the charge transfer process at the PTB7-Th/hPDI_2_-CN_2_ interface, which occurs within the instrument response time. The excitons at the PTB7-Th/hPDI_2_-CN_2_ interface quickly transform into polarons during the charge transfer process [43,44]. However, the excitons in the core of hPDI_2_-CN_2_ domain (E_C_) will undergo an ED process first before finally reach the D/A interface, followed by transformation into polarons through charge transfer process. Subsequently, the polarons will either turn into free carriers (FC) via a charge dissociation process or decay through a geminated recombination process. During this process, the population of FC would be reduced due to either bimolecular recombination (BR) process or being collected by electrode [45]. The rates of these processes and the corresponding spectra can be obtained from the global fitting results shown in figure 4*b*. For the DIO-treated device, the ED rate is 0.385 ps^−1^ while the geminate recombination rate is 0.0055 ps^−1^. As shown in figure 4*c*, the polaron signal retains even after the geminate recombination process (greater than 1000 ps). Therefore, the BR rate is very slow in the DIO-treated device, which is beneficial for the charge collection and the PCE improvement.

To explain the different PCEs of the three devices, the TA results of these three devices were carefully investigated. As shown in figure 4*d*, for the as-cast devices, the distinctive feature is related to the ED process. The signal of hPDI_2_-CN_2_ exciton (approx. 560 nm region), even at the initial time, is very weak. Taking into account of the morphology differences, most excitons of the as-cast blend are located at the PTB7-Th/hPDI_2_-CN_2_ interface. As shown in figure 4*c*, although the loss of excitons is reduced during the ED process, the rate of BR is much faster than the DIO-treated device. In this case, the BR is a major exciton depopulation channel rather than the ED process. As shown in figure 4*d*, similar to the situation of the as-cast device, fast BR process is observed for thermal-annealed device. By contrast, the slowest BR process is achieved for the DIO-treated device, and this may account for its best photovoltaic performance.

## 4. Conclusion

In summary, a novel hPDI molecule, hPDI_2_-CN_2_, was successfully synthesized and carefully characterized. The helical conformation of hPDI_2_-CN_2_ results in reduced molecular aggregation and intramolecular interactions. Cyclic voltammetry showed that the energy levels of hPDI_2_-CN_2_ were well matched with the low-bandgap donor, PTB7-Th, and they exhibit complementary absorption in the range of 300–800 nm. Microscopic morphologies of the blend films were carefully optimized with various treatments to improve the device performance. In comparison to the as-cast device with a PCE of 1.42%, an enhanced PCE of 2.76% was obtained for the thermal-annealed device. Furthermore, enabled by 0.5% DIO treatment, the PCE of device was significantly further increased to 3.25% with *V*_oc_ of 0.545 V, *J*_sc_ of 9.77 mA cm^−2^ and FF of 61.1%. The TA results demonstrated that a combination of fast ED rate and the lowest BR rate was achieved for DIO-treated blend film, thus leading to the best photovoltaic performance. This result provides further proof that the molecular conformation plays an important role in the design of PDI-based acceptors for OSCs.

## Supplementary Material

Supporting information

## Data accessibility

This article does not contain any additional data.
