# Supplementary material for: A helical perylene diimide-based acceptor for non-fullerene organic solar cells: synthesis, morphology and exciton dynamics
Source: R Soc Open Sci. 2018 May 2;5(5):172041. doi: 10.1098/rsos.172041 (PMC5990788; doi:10.1098/rsos.172041)
Supplement: Supporting information [file rsos172041supp1.docx]

**Electronic Supplementary Information (ESI) for**

**A Helical Perylene Diimide-based Acceptor for Non-Fullerene Organic Solar Cells: Synthesis, Morphology, and Exciton Dynamics**

Li Chen,^‡a,b^ Mingliang Wu,^‡a,b^ Guangwei Shao,^a^ Jiahua Hu,^a^ Guiying He,^a^ Tongle Bu,^b^ Jian-Peng Yi,*^a^ and Jianlong Xia*^a,b^

^a^School of Chemistry, Chemical Engineering and Life Science

^b^State Key Laboratory of Advanced Technology for Materials Synthesis and Processing

Wuhan University of Technology

No. 122 Luoshi Road, Wuhan 430070, China

^*^E-mail: Jian-Peng_Yi@outlook.com; jlxia@whut.edu.cn

‡These authors contributed equally to this work


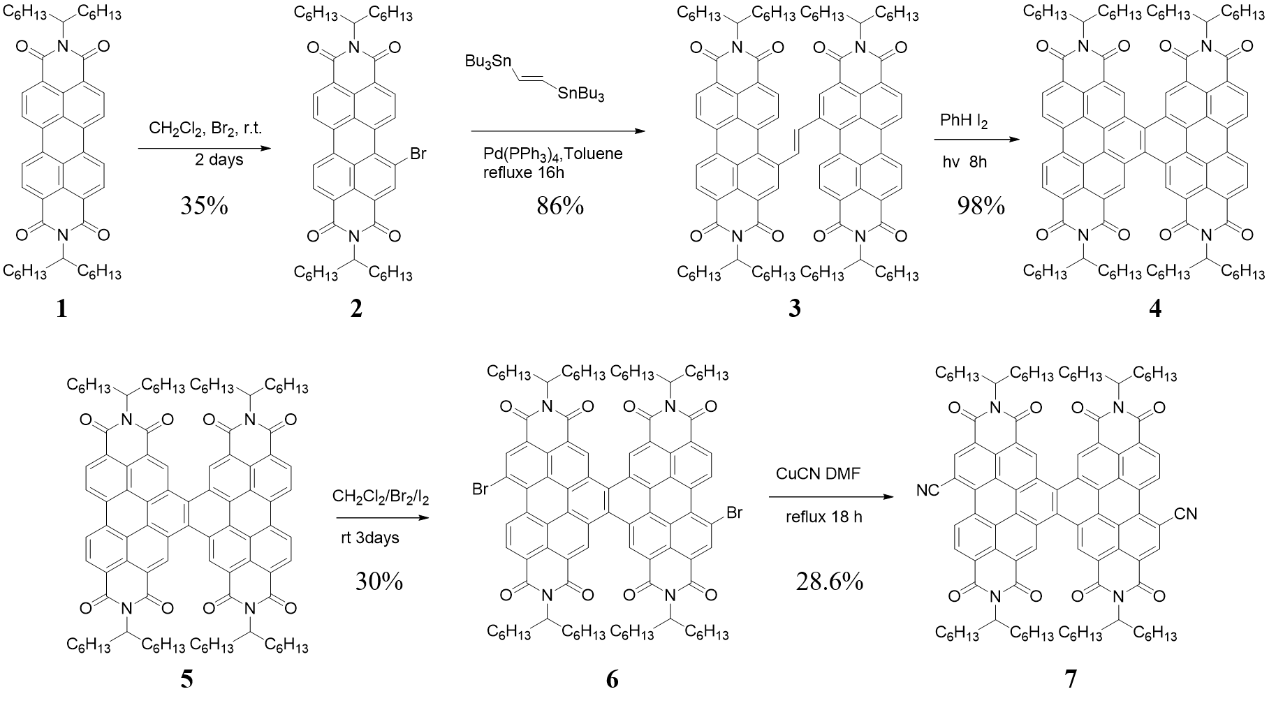


**Scheme S1**. The synthesis route of hPDI_2_-CN_2_.

Synthesis of hPDI_2_-CN_2_: The compound, hPDI_2_-Br_2_, was synthesized according to those previously reported.^1,2^ 1.5 g hPDI_2_-Br_2_ (0.89 mmol) and 0.9 g CuCN (10.12 mmol) was carefully mixed into a 250 mL-round bottom flask. Then the flask was degassed by N_2_ gas for 30 minutes. Next, 100 mL DMF was added by syringe and refluxed for 18 h. The mixture was cooled to room temperature and then poured into 200 mL water and stirred overnight. Further, the obtained product was extracted with 200 mL DCM and then washed with 300 mL water for three times. After removing the residual solvent, the crude product was purified through a silica chromatography (PE:DCM=1:2) and around 400 mg red-purple solid was obtained with a yield of 28.6 %. The alkyl chains linked with the imide nitrogen atoms are very beneficial to increase the solubility of the molecule in common organic solvents, such as toluene, chlorobenzene, and dichlorobenzene at room temperature. ^1^H NMR (400 MHz, Chloroform-d) δ 10.68 (d, J = 8.4 Hz, 2H), 10.35 (s, 4H), 9.49 (s, 2H), 9.32 (s, 2H), 5.32 (s, 4H), 2.35 (s, 8H), 1.98 (s, 8H), 1.31 (d, J = 42.4 Hz, 66H), 0.83 (s, 25H). ^13^C NMR (101 MHz, CDCl3) δ 163.45, 138.22, 135.56, 131.68, 131.51, 130.49, 128.99, 127.71, 127.23, 126.87, 126.57, 125.62, 125.50, 125.19, 125.06, 124.48, 120.27, 107.94, 55.62, 55.45, 32.44, 31.77, 29.24, 27.02, 22.62, 14.09. 13C NMR (101 MHz, CDCl3) δ 163.45, 138.22, 135.56, 131.68, 131.51, 130.49, 128.99, 127.71, 127.23, 126.87, 126.57, 125.62, 125.50, 125.19, 125.06, 124.48, 120.27, 107.94, 55.62, 55.45, 32.44, 31.77, 29.24, 27.02, 22.62, 14.09.


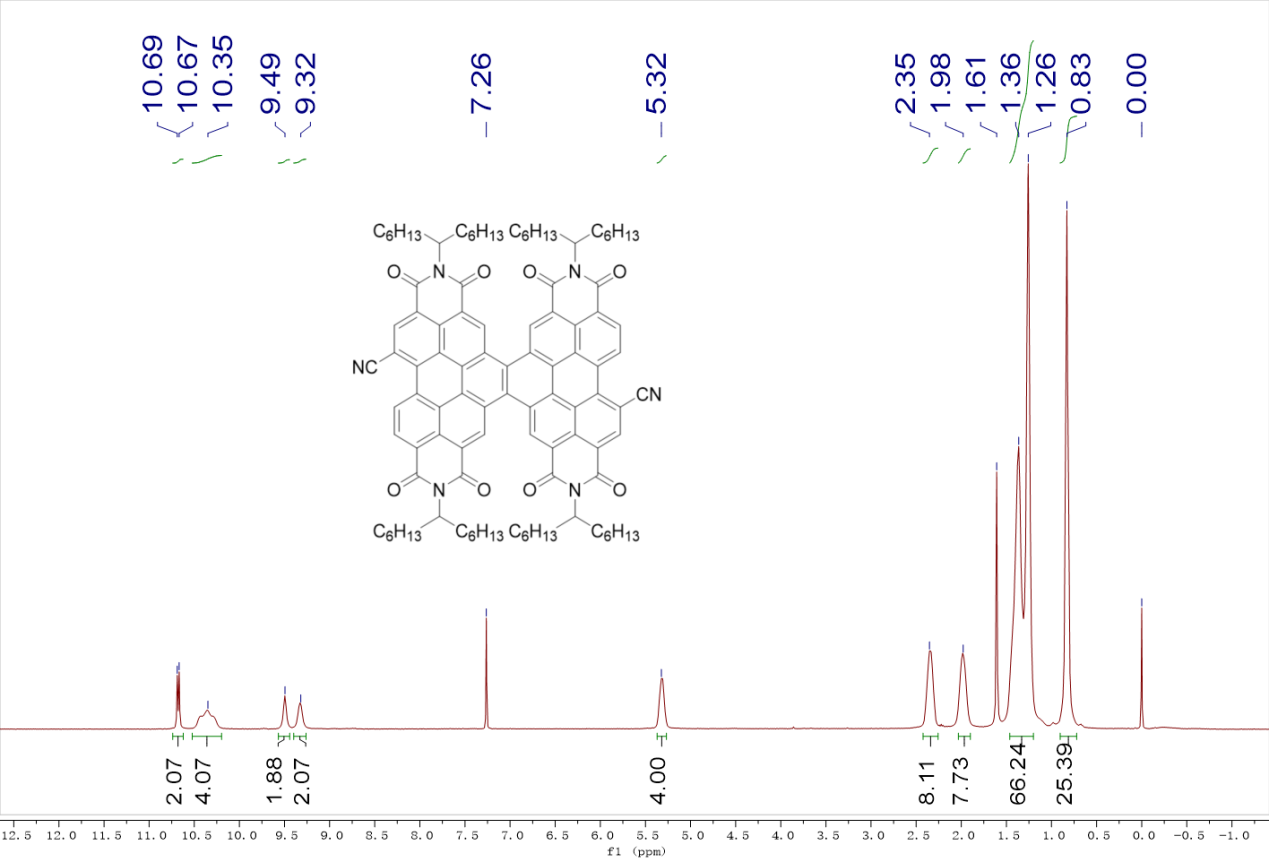


**Fig. S1**. ^1^H NMR spectrum of hPDI_2_-CN_2_.

**Fig. S2**. FTIR spectrum of hPDI_2_-CN_2_. The peak at 2220.2 cm^-1^ confirm the existence of cyano group.


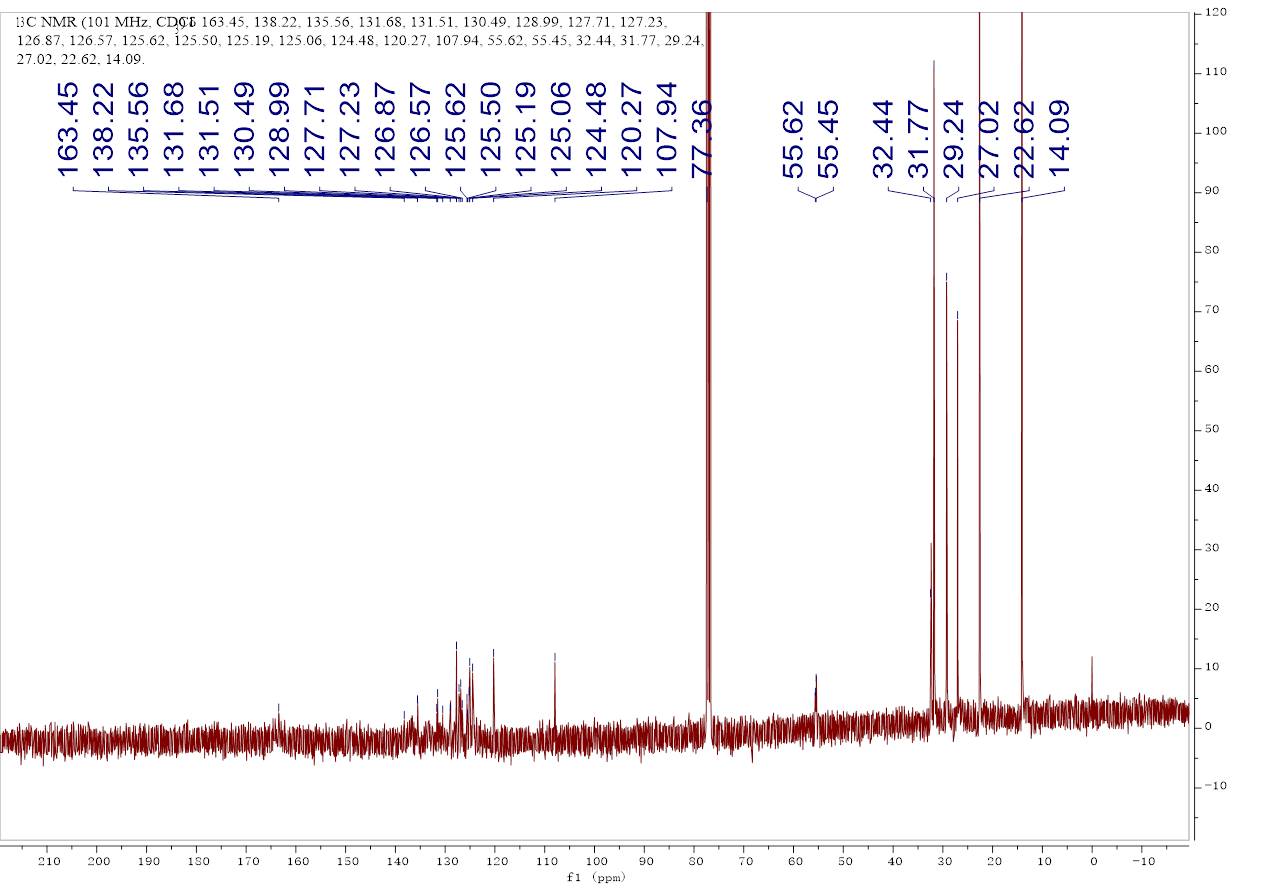


**Fig. S3**. 13C NMR spectrum of hPDI_2_-CN_2_.


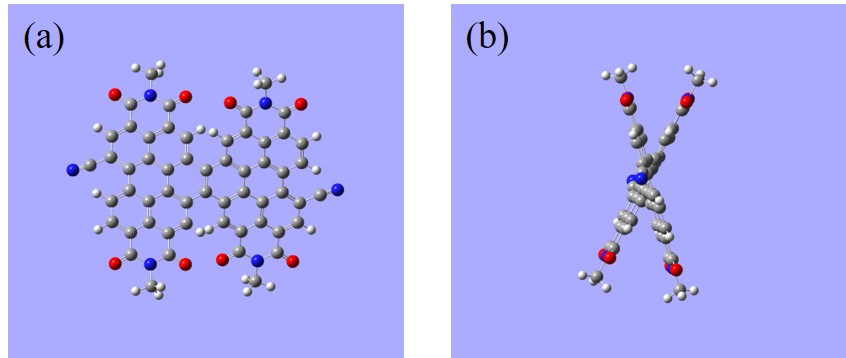


**Fig. S4**. DFT-calculated molecular structures of hPDI_2_-CN_2_: (a) top-view; (b) side-view.


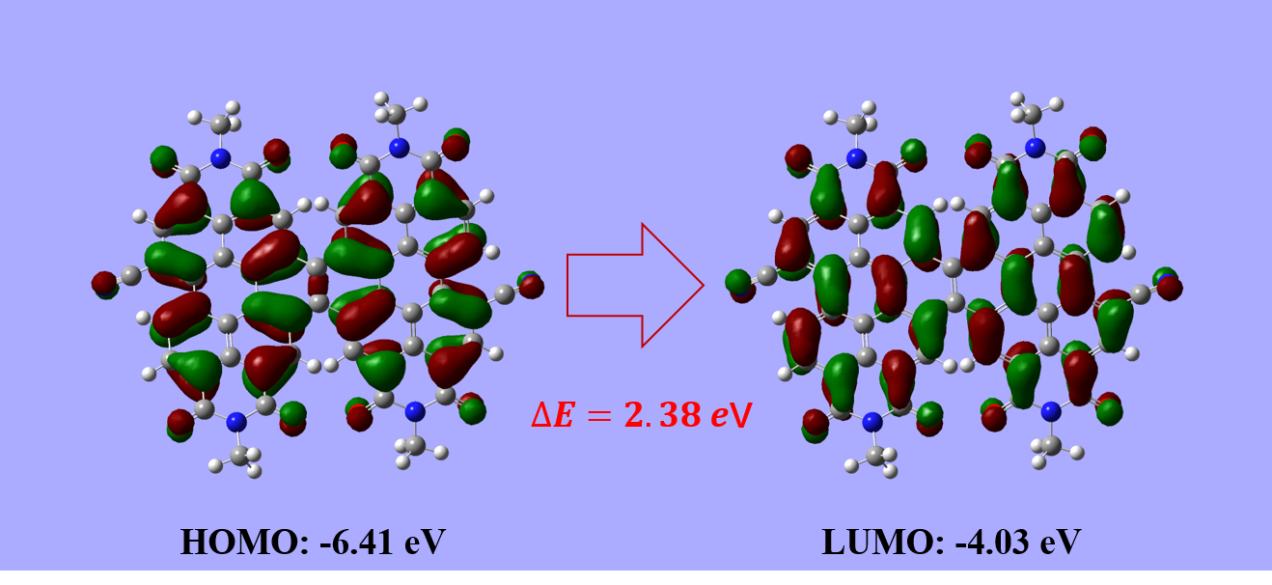


**Fig. S5.** Energy level diagrams and molecular configuration of hPDI_2_-CN_2_ simulated by DFT calculations.

**Fig. S6.** The cyclic voltammogram (CV) for hPDI_2_-CN_2_ in dichloromethane solution with a scanning rate of 200 mV/s.

**Table S1**. The electronic properties of hPDI_2_-CN_2_.

| Compound | *E*_HOMO_/eV^[a]^ | *E*_LUMO_/eV^[a]^ | *E*_g_/eV^[b]^ | *E*_g_^opt^/eV^[c]^ |
| --- | --- | --- | --- | --- |
| hPDI_2_-CN_2_ | -6.11 | -3.98 | 2.13 | 2.13 |

^[a]^Estimated from the onset oxidation and reduction potential by using *E*_HOMO_ = *E*_LUMO_-*E*_g_^opt^eV and*E*_LUMO_ *=* - (*E*_red_ - *E*_Fc_ + 4.8) eV , *E*_Fc/Fc+_=0.57 eV. ^[b]^*E*_g_= *E*_LUMO_- *E*_HOMO_. ^[c]^Calculated from the absorption spectrum of hPDI_2_-CN_2_ film according to the equation of $E_{g}=1240/\lambda$_onset_.

**Fig. S7.** The TGA result of hPDI_2_-CN_2_.

**Fig. S8.** The *J*-*V* curves for OSCs with various thermal annealing temperatures.

**Table S2.** Summary of device parameters with a D-A mass ratio of 1:1.5 upon different annealing temperatures.

| Annealing Temperature/^o^C | *V*_oc_/V | *J*_sc_/mA cm^-2^ | FF/% | PCE/% |
| --- | --- | --- | --- | --- |
| 80 | 0.461 | 8.79 | 38.3 | 1.55 |
| 100 | 0.498 | 8.68 | 51.8 | 2.24 |
| 120 | 0.495 | 8.91 | 52.3 | 2.31 |
| 140 | 0.493 | 8.98 | 53.9 | 2.39 |
| 160 | 0.488 | 9.68 | 51.9 | 2.45 |
| 180 | 0.477 | 8.70 | 49.5 | 2.05 |


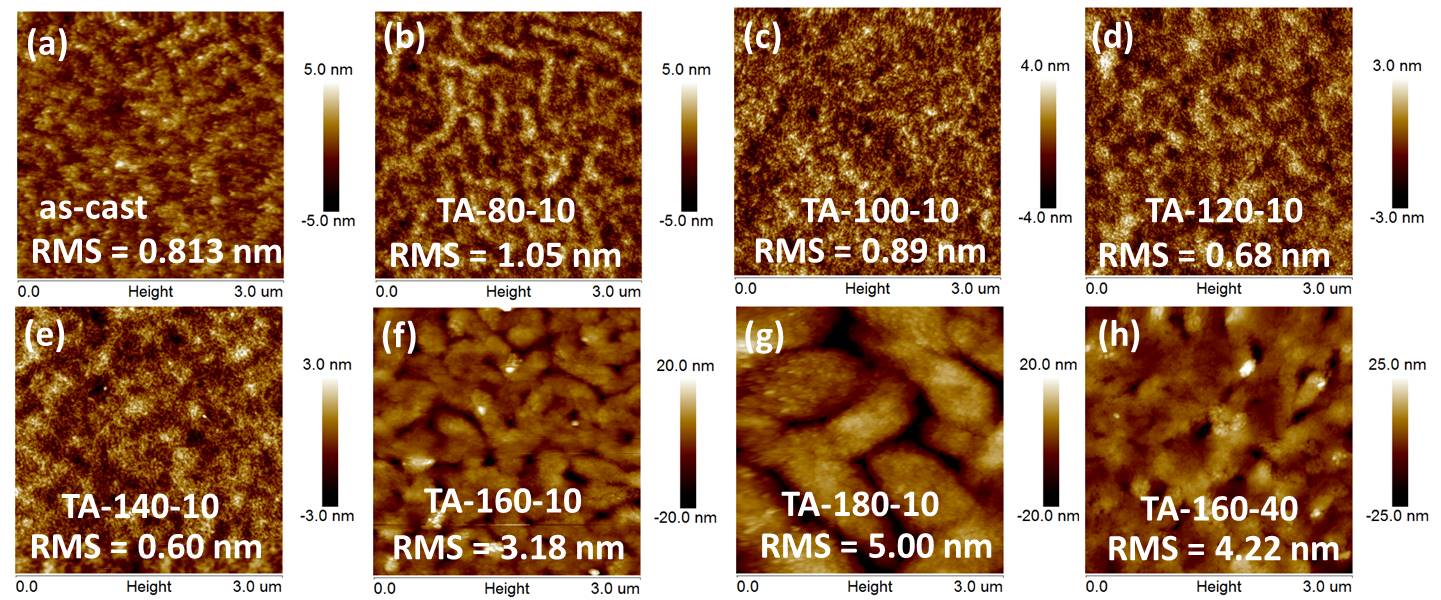


**Fig. S9**. Atomic force microscope (AFM) images for the PTB7-Th:hPDI_2_-CN_2_ blending films with different treatments .


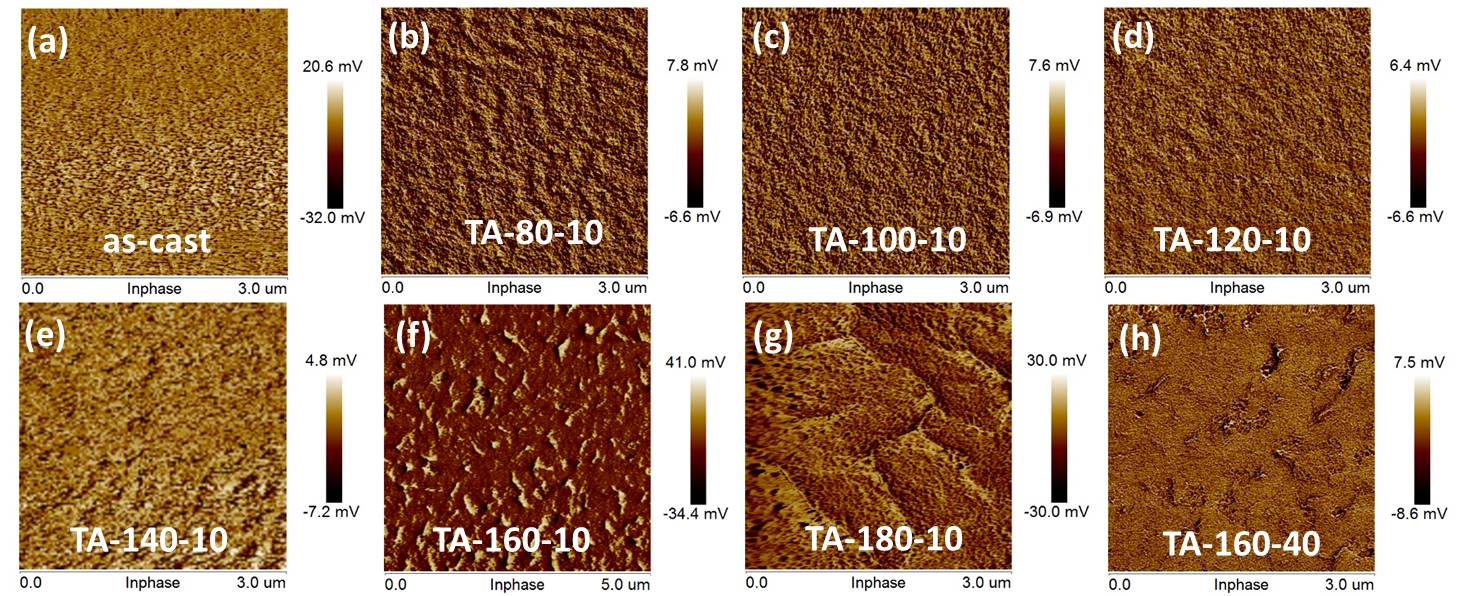


**Fig. S10**. AFM phase images for the PTB7-Th:hPDI_2_-CN_2_ blending films based on different treatments.

**Fig. S11**. The *J*-*V* curves for the devices with different D-A ratios annealing upon 160 ^o^C for 40 min.

**Table S3**. Summary of device performances with different D-A ratios.

| PTB7-Th:hPDI_2_-CN_2_ | *V*_oc_/V | *J*_sc_/mA cm^-2^ | FF/% | PCE/% |
| --- | --- | --- | --- | --- |
| 1:1 | 0.492 | 9.11 | 42.7 | 1.91 |
| 1:1.5 | 0.505 | 9.04 | 57.4 | 2.62 |
| 1:2.0 | 0.506 | 8.82 | 56.7 | 2.53 |
| 1:2.5 | 0.506 | 9.29 | 58.7 | 2.76 |
| 1:3.0 | 0.521 | 8.59 | 59.2 | 2.65 |
| 1:4.0 | 0.526 | 7.91 | 63.3 | 2.64 |

**Fig. S12**. The *J*-*V* curves for the OSCs with different additive treatments.

**Table S4**. Performance parameters for the devices based on different additive treatments.

| Additive | *V*_oc_/V | *J*_sc_/mA cm^-2^ | FF/% | PCE/% |
| --- | --- | --- | --- | --- |
| DMF | 0.509$\pm$0.03 | 9.04$\pm$0.3 | 54.5$\pm$3 | 2.51 (2.85) |
| NMP | 0.498$\pm$0.03 | 9.08$\pm$0.2 | 57.3$\pm2$ | 2.59 (2.76) |
| CN | 0.516$\pm$0.02 | 9.18$\pm$0.2 | 62.3$\pm1$ | 2.95 (2.99) |


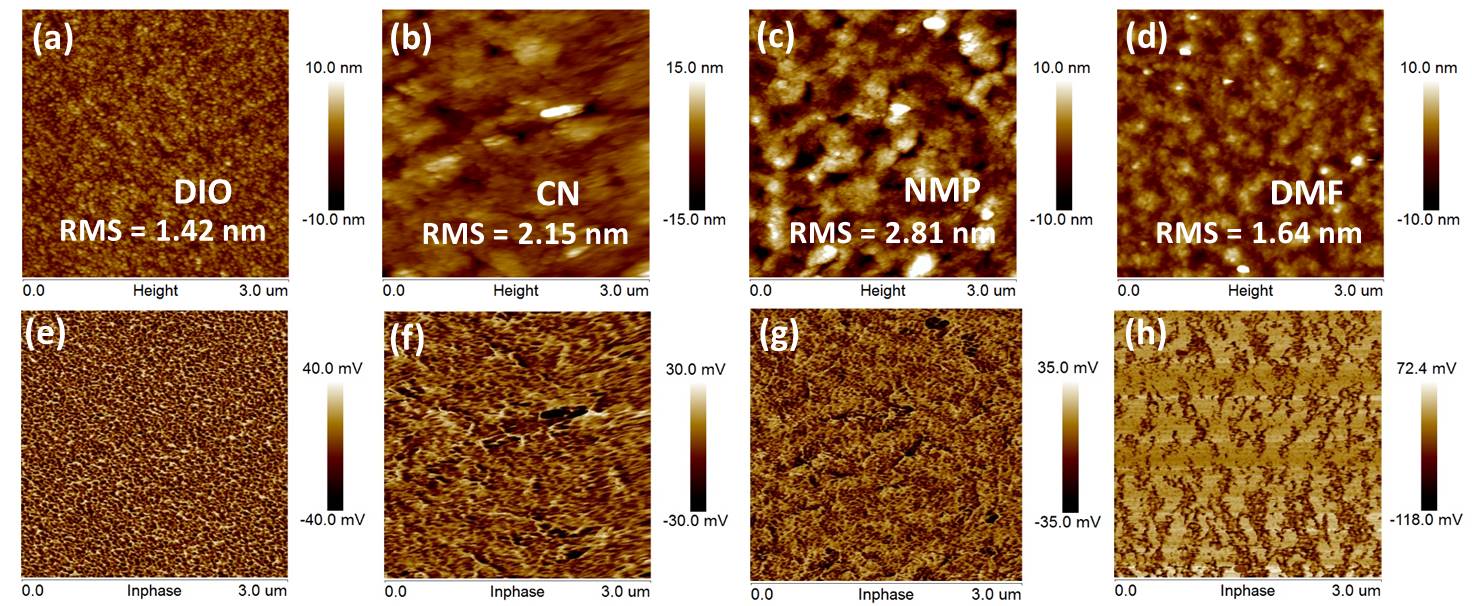


**Fig. S13**. The AFM height (a, b, c, d,) and corresponding phase (e, f, g, h) images for DIO, CN, NMP, DMF-treated PTB7-Th:hPDI_2_-CN_2_ blending films, respectively.

**Fig. S14**. The J-V curves of the devices based on different DIO ratios.

**Table S5**. Summary of device performance of organic solar cells with different DIO amounts

| DIO ratio/% | *V*_oc_/V | *J*_sc_/mA cm^-2^ | FF/% | PCE/% |
| --- | --- | --- | --- | --- |
| 0 | 0.506 | 9.29 | 58.7 | 2.76 |
| 0.5 | 0.545 | 9.77 | 61.1 | 3.25 |
| 1.0 | 0.517 | 8.82 | 61.0 | 2.78 |
| 1.5 | 0.540 | 5.82 | 48.8 | 1.53 |
| 2.0 | 0.518 | 7.05 | 56.7 | 2.07 |
| 3.0 | 0.523 | 8.12 | 59.6 | 2.53 |

**Fig. S15**. DIO-treated device parameters *V*_oc_, *J*_sc_ (a) and FF, PCE (b) as a function of annealing time length.


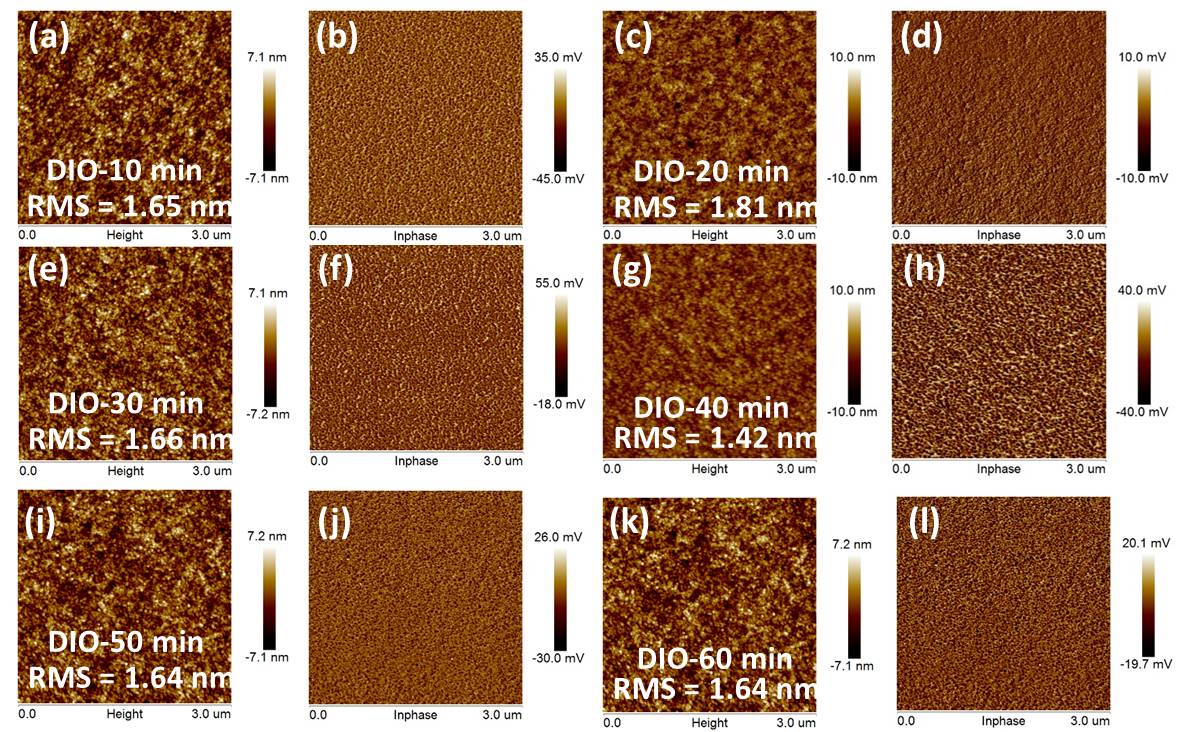


**Fig. S16**. The AFM height (a, c, e, g, i, k) and corresponding phase (b, d, f, h, j, l) images of DIO-treated PTB7-Th:hPDI_2_-CN_2_ blending films with various annealing time lengths upon 160 ^o^C.

**Fig. S17.** The photoluminescence (PL) spectra for pure PTB7-Th film (solid squares) and the PTB7-Th:hPDI_2_-CN_2_ (1:2.5 w/w) blending films with various treatment as-cast (solid up-triangles), traditional annealing at 160 ^o^C for 40 min (solid up-side down triangles), and 0.5% (vol%) DIO treatment (solid spheres).

**Fig.S18.** *J*^1/2^-*V* plots and the corresponding linear fittings for PTB7-Th:hPDI_2_-CN_2_ (1:2.5, w/w)-based hole-only (a) and electron-only devices (b).

**Table S6**. Summary of average charge carrier mobility values determined by SCLC methods.

| Treatment | *μ*_h_(cm^2^ V^-1^S^-1^) | *μ_e_*(cm^2^ V^-1^S^-1^) | *μ*_h/_*μ*_e_ |
| --- | --- | --- | --- |
| as-cast | 6.0$\times$10^-5^ | 9.0$\times$10^-5^ | 0.67 |
| TA-160 ^o^C - 40 min | 1.9$\times$10^-4^ | 1.6$\times$10^-3^ | 0.12 |
| 0.5% DIO | 4.3$\times$10^-4^ | 4.0$\times$10^-4^ | 1.08 |


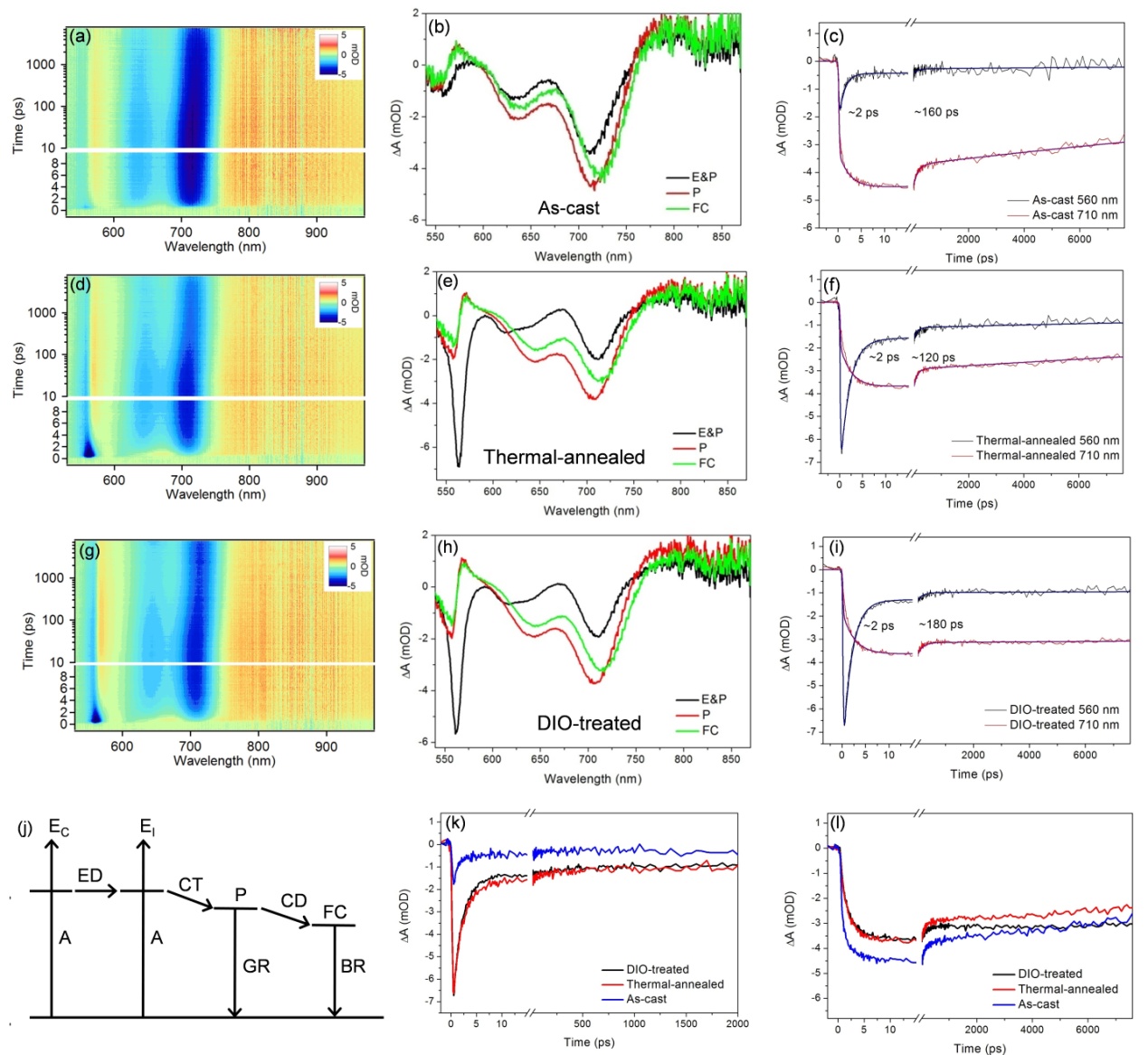


**Fig. S19.** (a) The TA result shown in a pseudo-color plot, (b) global fitting results and (c) representative kinetics of as-cast device. (d) The TA result shown in a pseudo-color plot, (e) global fitting results and (f) representative kinetics of thermal-annealed device. (g) The TA result shown in a pseudo-color plot, (e) global fitting results and (f) representative kinetics of DIO-treated device. (j) The photovoltaic conversion process. (k) The representative kinetics at 560 nm for the three devices. (l) The representative kinetics at 710 nm for the three devices.

**References**

1 Y. Zhong, M. T. Trinh, R. Chen, W. Wang, P. P. Khlyabich, B. Kumar, Q. Xu, C. Y. Nam, M. Y. Sfeir and C. Black, *J. Am. Chem. Soc.*, 2014, **136**, 15215.

2 Y. Zhong, M. T. Trinh, R. Chen, G. E. Purdum, P. P. Khlyabich, M. Sezen, S. Oh, H. Zhu, B. Fowler and B. Zhang, *Nat. Commun.*, 2015, **6**, 8242.
